# Evaluation of Lateral Crura Divergence Angle of Iranian Candidates for Primary Rhinoplasty

**DOI:** 10.29252/wjps.10.1.3

**Published:** 2021-01

**Authors:** Shahriar Loghmani, Shirin Loghmani, Mehri Doosti Irani, Mohammadreza Zarei, Fatemeh Maraki

**Affiliations:** 1 Department of Plastic Surgery, Ordibehesht Surgical Center, Isfahan, Iran;; 2 Assistant Professor, School of Nursing and Midwifery, Research Center in Midwifery and nursing Sciences, Shahrekord University of Medical Sciences, Shahrekord, Iran;; 3 Student Research Committee, School of Nursing and Midwifery, Isfahan University of Medical Sciences, Isfahan, Iran;; 4 Shahrekord University of Medical Sciences, Shahrekord, Iran.

**Keywords:** Rhinoplasty, Lateral crura divergence angle, Nasal tip, Cephalic malposition, Iran

## Abstract

**BACKGROUND:**

Cephalic malposition of the lower lateral cartilages is a common nasal anatomic variation. Knowing the range of lateral crura (LC) divergence angle in Iranian population can help Middle East plastic surgeons. This study aimed to determine LC divergence angle of candidates for primary rhinoplasty in Iranian population.

**METHODS:**

This cross-sectional study was conducted on 256 candidates for primary rhinoplasty from November 2017 through May 2018. Two sides of LC divergence angle were measured intraoperatively by a researcher-made device.

**RESULTS:**

Totally, 211 female and 45 male patients with the mean age of 29.9±6.51 years were recruited. The mean LC divergence angle was 35.86±4.74° (between 20-50°). The mean LC divergence angle was 35.11° and 36.02° in male and females, respectively. There was no significant difference between males and females. In addition, there was no significant correlation between LC divergence angle and age. LC divergence angle had normal distribution and about 68% of the LC divergence angle were within one standard deviation of the mean (i.e. 32 to 40 degree).

**CONCLUSION:**

In 16% of studied people, the divergence angle of the lateral crus of the lower lateral cartilage was lower than 32° and was considered as malposition.

## INTRODUCTION

The rhinoplasty initially aimed to create an attractive, functional nose without any surgical stigmata.^1^^-^^3^ Having a detailed knowledge about nasal anatomy and comprehensive analysis of the facial and nasal region is important for surgeons to achieve the excellent results in rhinoplasty. In addition, preoperative planning is important for plastic surgeons to prevent unpredictable results.^4^ There are some techniques and maneuvers for achieving a more beautiful nose. Facial features and anthropometric parameters of the patient’s nose can determine the optimal maneuvers.^5^ There are some anatomic variations in the nose. Although there are different definitions for malposition, but one of the most common definitions is based on the angle of the lateral crura (LC) and the midline.^6^ LC divergence angle (LCDA) is a common anatomic variation. Small LCDA can cause a condition called “malposition”.^7^ Shape of nasal tip is affected by malposition and support of alar rim constitution.^8^ Cephalic malposition affects external features, such as nasal tip shape and alar rim, as well as the nasal obstruction results.^9^ Various maneuvers are considered specifically to correct this abnormality, such as composite grafts, and repositioning, or even cartilage Z-plasty.^10^ Therefore, it is necessary to know the normal range of LCDA to obtain the best aesthetic results. Based on previous report, the angle of the cephalic-positioned LC and midline was mentioned 30 degrees or less.^8^ Various authors have cited different numbers for LCDA-based malpositioning and a definitive classification has not been established.^11^ While attempts have been made to know nasal parameters in different societies,^12^ the normal range of LCDA is not determined for Iranian population. This study aimed to determine LC divergence angle of Iranian candidates for primary rhinoplasty. This seems to be important and it can be useful for Middle East plastic surgeons to choose the best maneuvers and techniques to improve the outcomes of rhinoplasty.

## MATERIALS AND METHODS

**Fig. 1:**
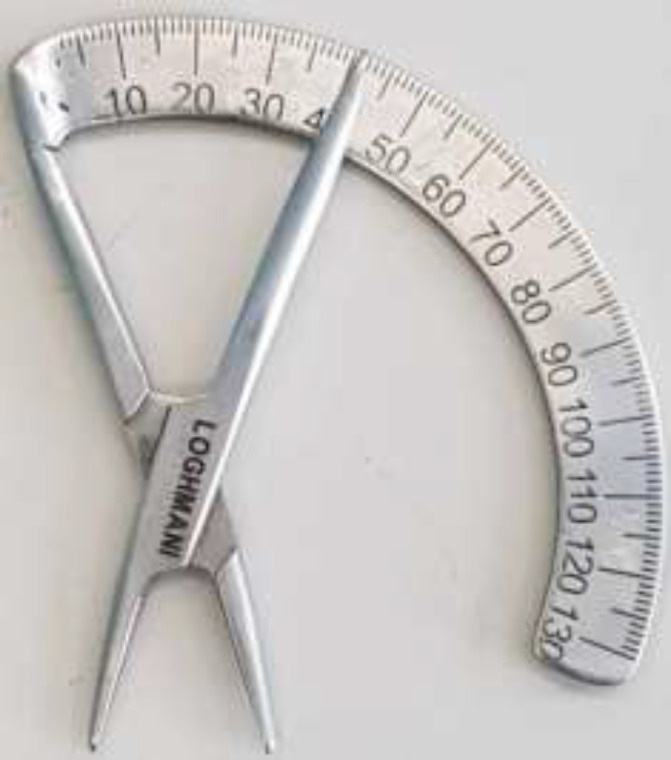
Researcher-made device for measuring LCDA

This Cross-sectional study was conducted in a private surgical center from November 2017 to May 2018. Iranian candidates for primary rhinoplasty were informed about the research and finally 256 patients were recruited through the convenient sampling method. They signed a written informed consent form. People who had history of serious nasal injuries, previous nose surgery, congenital facial abnormalities, and major septal deviation were excluded. Data collection was done through a researcher-made device measuring LCDA (Figure 1), and the demographic questionnaire (including age and sex). 

All patients underwent primary open rhinoplasty under general anesthesia. Local anesthesia (2% lidocaine and 1:80,000 adrenaline) was also infiltrated into the incision and dissection planes. A stair-step columellar incision and bilateral marginal incisions were made, and the nasal flap was elevated in the subperichondrial plane. 

**Fig. 2:**
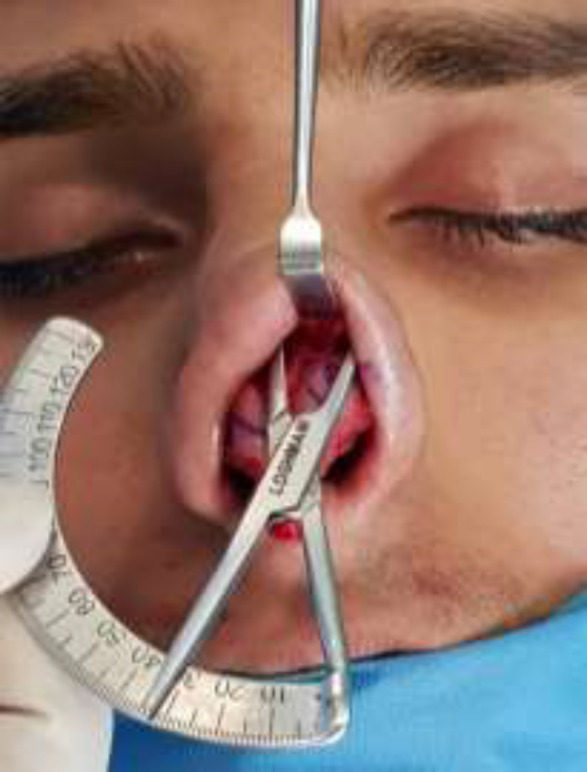
Intraoperative LCDA measuring

Measuring LCDA was done after skeleonization, and before cephalic resection of the LC and cutting intercrural and intradomal ligaments. For this purpose, the middle point of medial portion of LC was marked with ink, and then the middle point of lateral portion of LC (near A1 junction) was marked. These two points were connected with one line together. Finally, the angle of each line was measured relative to the midline and theLCDA on the right and left of the nose was determined (Figure 2).

Statistical analyses were performed using SPSS software (version 20.0, IBM Corp, Armonk, NY, USA). Descriptive statistics of continuous variables were represented with the mean, standard deviation, median, minimum, and maximum values. The Pearson’s correlation analysis was used to examine the correlations of the LCDA with the patients’ age. The Student’s t-test was conducted for comparing the mean of LCDA between men and women. Statistical significance was defined as *p*<0.05.

## RESULTS

This study was undertaken on 256 patients, while most of them (n=211) were female (82.4%). The age ranged from 18 to 52 years, with a mean (SD) of 29.9±6.51 years. Given that in all 256 patients, each LCDA was measured relative to the midline (septum) independently, and the total 512 LCDA was achieved. Mean (SD) LCDA was 35.86±4.74 degrees (ranged between 20 to 50 degrees). Independent t-test (Table 1) showed that the mean LCDA was not significantly different between men and women (*p*>0.05). Pearson correlation coefficient (Table 2) showed that there was no significant relationship between LCDA and subjects’ age (*p*>0.05). 

**Fig. 3:**
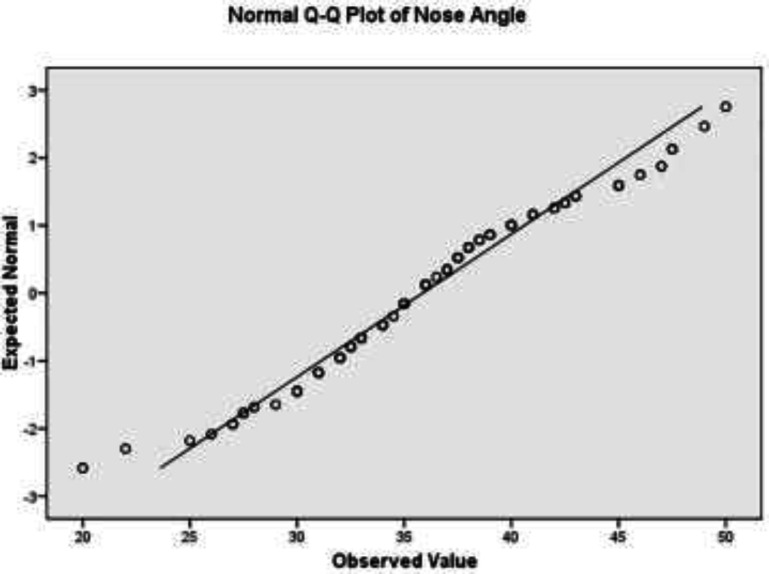
Q-Q diagram to check normality of nose angle distribution

**Fig. 4:**
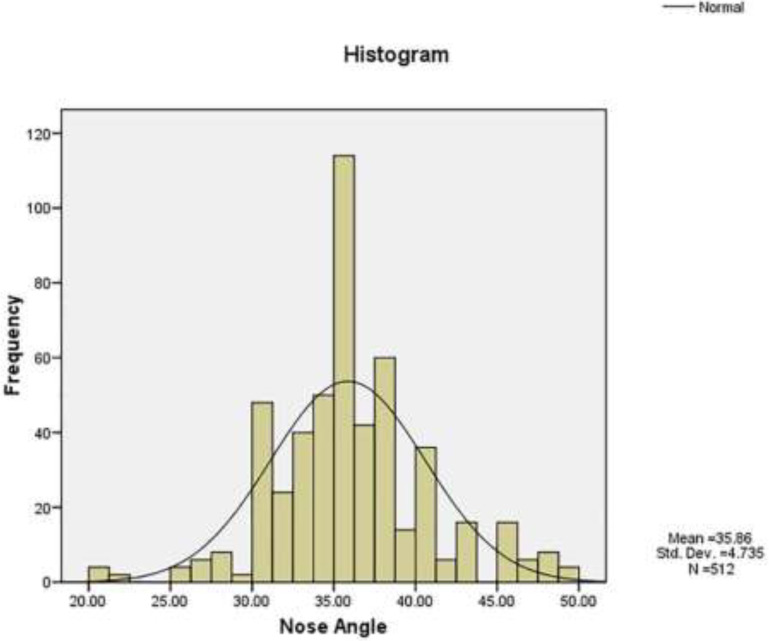
Frequency distribution of the nasal angle and its fitting to the normal distribution

Figures 3 and 4 shoes that the LCDA had a normal distribution. Figure 2 and obtained 2.5^th^ and 97.5^th^ percentiles for LCDA showed that the angle was 27 to 47.1 degrees in 95% of the studied subjects. In addition, the 16^th^ and 84^th^ percentiles of LCDA were 32 and 40 degrees, respectively. About 68 percent of the LCDA were within one standard deviation of the mean (i.e. 32 to 40 degrees); and about 95 percent of the LCDA were within two standard deviation of the mean (i.e. 26 to 45 degrees); and finally, about 99 percent of the LCDA were within three standard deviation of the mean (i.e. 22 to 49 degrees). 

**Table 1:** LCDA according to sex

**Variable**	**Male**			**Female**			**Independent t test**	
	**Mean±SD**	**Min**	**Max**	**Mean±SD**	**Min**	**Max**	**T**	*p*
LCDA	35.11±4.71	20	47.5	36.02±4.73	20	50	1.65	0.10

LCDA: Lateral crura divergence angle

**Table 2:** Pearson correlation coefficients between LCDA and age

**Variable**	**Age**	
	**R**	**P**
LCDA	-0.012	0.79

LCDA: Lateral crura divergence angle

## DISCUSSION

LCDA of 256 Iranian candidates for primary rhinoplasty was measured and 512 LCDA were enrolled. According to the results, their mean age (SD) was 29.9±6.51 years and 82.4% were female. Mean (SD) LCDA was 35.86±4.74 degrees (ranged between 20 to 50 degrees). Mean (SD) LCDA was 35.11 and 36.02 degrees in males and females, respectively. There was no different significance between men and women and also in relation to age (p>0.05). 72 Iranian cadavers with the mean age of 42 years were studied and shown that 62% were male and the mean (SD) LCDA was 38.6±3.9 degrees, while the mean LCDA was 39.45 and 37.2 degrees in males and females, respectively with no significance differences.^12^ These findings are similar to our study due to Iranian subjects recruited in their study.

Studying 40 patients who were Caucasian females with mean age of 28 years undergoing rhinoplasty demonstrated a LCDA between 30 to 60 degrees with a mean of 43.6 degrees.^13^ The study population affected a difference in the results with our findings. In a study carried out to determine the anthropometric changes of nose, an increase in age of 70 candidates for cosmetic rhinoplasty was illustrated. The LLC was the nasal tip supportive mechanism that was mostly affected by age as a year of increase resulted to a weakening of −1.077% degrees in LLC. However, this study has not examined changes in LCDA with aging.^14^

According to another report, the LC malposition was very common as it occurred in approximately 50% of primary rhinoplasties and in many of secondary rhinoplasties too (more than 80%) and also in “ball” or “box” tips and on the cleft side of deformities in the cleft lip nasal.^10^ Therefore, we determined the normal range of LCDA in the Iranian population and the angle of malposition too. On the other hand, the definition of LCDA in different races and communities can be different due to the anatomical variations of different races. In addition, accurate estimation of malposition based on the nose appearance (preoperative) was not possible for patients who had pseudomalposition associated with the boxy pinch. Therefore, determining LCDA can provide more accurate definition of the malposition. The results of our study showed that LCDA had a normal distribution and about 68 percent of the LCDA were within one standard deviation of the mean (i.e. 32 to 40 degrees). So LCDA that was less than 32 degrees was considered malposition in the Iranian community.

As already described in the literature, the orthotopic position of the LC lies between 30° and 45°, and cephalic malposition is defined by LC angles of <30° from the midline septal plane.^15^ Several studies introduced LCDA equal to or less than 30 degrees relative to midline as malposition.^16^^-^^18^ Therefore, there was no definitive definition for LCDA in the Iranian population. The results of our study may be useful for Middle East surgeons, because they can be more confident in identifying the complication and use appropriate techniques and maneuvers to address it. We acknowledge some limitation in this study too. Studies with larger population may provide more reliable data about evaluation of LCDA of candidates for primary rhinoplasty in Iranian population.

## CONCLUSION

Cephalic malposition of the lower lateral cartilages (LLD) is a frequent anatomic variation of nasal anatomy. In the Iranian population, LCDA follows the normal distribution and in 68% of people, the divergence angle of the LC of the LLC is between 32° and 40° from the midline.

## CONFLICT OF INTEREST

The author has no conflicts of interest to declare.
